# Exploring the efficacy of acupuncture for tension-type headache: a literature review and insights from traditional Chinese medicine

**DOI:** 10.22514/jofph.2024.035

**Published:** 2024-12-12

**Authors:** Hao Wang, Hongyuan Chang, Anmin Wang, Dicheng Luo, Chen Huang, Jianfeng Huang, Jiangwei Zhang, Xiangping Sun

**Affiliations:** ^1^Xiyuan Hospital of China Academy of Chinese Medical Sciences, 100091 Beijing, China; ^2^Ningxia Traditional Chinese Medicine Hospital and Research Institute of Traditional Chinese Medicine, 750021 Yinchuan, Ningxia Hui Autonomous Region, China

**Keywords:** Acupuncture, Tension-type headache, Traditional Chinese medicine, Mechanism

## Abstract

Tension-type headache (TTH) is a common primary headache disorder, and recent research has focused on various treatment options. However, studies evaluating acupuncture for TTH from the perspective of Traditional Chinese Medicine (TCM) and its mechanisms are limited. This literature review synthesizes findings from twelve clinical studies that investigated acupuncture for TTH treatment. Data analysis was conducted for acupoint selection, types of acupuncture, treatment duration and needle retention time in these studies considering TCM principles. Our results indicate that acupuncture practitioners should select acupoints based on TCM syndrome differentiation and patient-specific factors. The optimal treatment duration is at least four weeks, with each session lasting a minimum of 20 minutes, and 30 minutes per session is recommended for enhanced efficacy. Additionally, the therapeutic effects of acupuncture on TTH may involve mechanisms such as the inhibition of myofascial trigger points and the modulation of central sensitization.

## 1. Introduction

Tension-type headache (TTH) is a prevalent primary headache disorder characterized by mild to moderate pressure or tension in the head [1, 2]. Its prevalence in the general population ranges from 30% to 78%, with a higher incidence observed in women compared to men [3, 4]. In China, the prevalence varies between 10.8% and 25.2% and is on the rise [5, 6]. TTH significantly impacts the disability-adjusted life years of young and middle-aged individuals [7], affecting mood, work performance and daily life. It not only diminishes quality of life and impairs physical and mental health [8] but also imposes a substantial economic burden on both society and families. Although current treatment options for TTH include antidepressants, these medications are often associated with adverse effects and poor tolerance, especially in elderly patients [9]. In addition, nonsteroidal anti-inflammatory drugs (NSAIDs) may cause gastrointestinal symptoms as well as liver and kidney toxicity [10].

Acupuncture, a significant component of Traditional Chinese Medicine (TCM), has demonstrated clinical efficacy and safety in managing various neurological disorders and pain-related conditions [11, 12]. Notably, its effectiveness in treating TTH is well-documented, with evidence indicating improvements in blood circulation in the head and neck region, a reduction in disease duration and a high level of patient satisfaction [13]. While previous reviews have addressed the efficacy and safety of acupuncture for TTH [14], they often lack detailed discussions on critical aspects such as acupoint selection, needle retention time, treatment duration, and underlying mechanisms.

In TCM, TTH is classified under the broader category of “headache”, with its earliest references found in The Yellow Emperor’s Classic of Internal Medicine (YECIM) scripts. According to YECIM, headaches are thought to result from external factors such as wind, cold, dampness and heat or from internal imbalances including dampness, heat, blood stasis, weakness and qi stagnation, which disrupt the overall balance of yin and yang in the body [15]. Ancient Chinese practitioners integrated theoretical concepts with clinical experience to classify headaches into various TCM syndromes, characterized by specific headache symptoms, accompanying signs and diagnostic indicators such as tongue appearance and pulse quality [16]. The treatment approaches, including herbal remedies and acupuncture, differ based on the specific TCM syndrome diagnosed. Notably, Zhang Zhongjing, a prominent TCM physician of the Han Dynasty, introduced an alternative diagnostic framework in his Treatise on Febrile Diseases. He categorized headaches based on their location in the meridians—Taiyang, Yangming, Shaoyang and Jueyin—rather than focusing on accompanying symptoms and tongue and pulse characteristics [17].

Acupuncture and herbal therapies are both essential elements of TCM, with acupuncture specifically focusing on the selection and combination of acupoints, each having distinct therapeutic effects. Ancient TCM practitioners, despite differing in their choice of acupoints for headache treatment, based their acupuncture prescriptions on their clinical experience and aligned them with relevant TCM syndromes and meridian theories [17]. This review aims to address these gaps by providing a detailed analysis of these factors from both TCM and Western medicine perspectives and proposes a preliminary therapeutic regimen to assist clinicians in effectively utilizing acupuncture for the treatment of TTH.

## 2. Modern application and clinical trials on acupuncture in TTH

We conducted a systematic search of PubMed and Web of Science databases for relevant studies up to January 2024. Additionally, we reviewed the reference lists of related studies to identify any potentially eligible clinical trials. The search strategy is outlined in Table 1.

**Table 1.** Search strategy.

No.	Search items
#1	Acupuncture (Title/Abstract)
#2	Acupuncture treatment (Title/Abstract)
#3	Electroacupuncture (Title/Abstract)
#4	Acupuncture therapy (Title/Abstract)
#5	Acupuncture points (Title/Abstract)
#6	Acupuncture analgesia (Title/Abstract)
#7	Fire needling (Title/Abstract)
#8	Scalp acupuncture (Title/Abstract)
#9	Ear acupuncture (Title/Abstract)
#10	Or/#1–#9
#11	Tension-Type Headache (Title/Abstract)
#12	Tension Type Headache (Title/Abstract)
#13	Tension Headache (Title/Abstract)
#14	Psychogenic Headache (Title/Abstract)
#15	Tension-Vascular Headache (Title/Abstract)
#16	Tension Vascular Headache (Title/Abstract)
#17	Or/#11–#16
#18	Clinical trial (Publication Type)
#19	Clinical article (Publication Type)
#20	Clinical study (Publication Type)
#21	Randomized controlled trial (Publication Type)
#22	Or/#18–#21
#23	#10 and #17 and #22

The inclusion criteria included the following: (1) Participants: Adults (age ≥18) diagnosed with TTH according to International Headache Society criteria; (2) Intervention: Acupuncture therapy alone, without additional treatments; (3) Control: Comparisons to sham acupuncture, placebo, herbs or other control conditions (*e.g.*, waiting list, usual care, no treatment); (4) Outcomes: Responder rate (defined as a ≥50% reduction in TTH frequency), TTH frequency, pain intensity, headache duration, medication consumption and related adverse events; (5) Article Type: Clinical trials, including randomized controlled trials (RCTs), crossover RCTs, *etc.*, published in English. However, the following studies were excluded: (1) Nonclinical studies (*e.g.*, animal experiments, reviews, protocols, case reports, letters, comments); (2) Duplicates and studies with incomplete data; (3) Studies focusing on treatments other than acupuncture (*e.g.*, dry needling, moxibustion); (4) Studies not primarily addressing TTH (*e.g.*, migraine, uncertain diagnosis).

We retrieved a total of 793 articles from PubMed, Web of Science and related reference lists. After removing duplicates, 367 studies were included for assessment. Their titles and abstracts were screened, leading to the exclusion of 320 studies that were either not clinical studies or unrelated to acupuncture. A full-text review resulted in the exclusion of 35 additional studies, and the study selection chart is shown in Fig. 1.

**Fig. 1.** Selection procedures.

## 3. Results

A total of 12 English-language clinical studies involving 659 participants who received acupuncture, electroacupuncture or laser acupuncture were found eligible. Among these studies, 11 were randomized controlled trials (RCTs), and one was a crossover RCT [18, 19, 20, 21, 22, 23, 24, 25, 26, 27, 28, 29]. Of the 12 studies, 6 focused on chronic TTH [18, 20, 21, 23, 25, 28], while the remaining 6 concentrated on mixed TTH [19, 22, 24, 26, 27, 29].

Regarding the selection of acupoints, there was considerable variation among the 12 studies. Chassot *et al.* [20] did not specify the names of the acupoints used despite employing acupuncture therapy. Tavola *et al.* [29] provided only approximate descriptions of the acupoints’ locations, such as on the head, hands or feet. Interestingly, many studies incorporated the principles of TCM differentiation by tailoring acupoint selection to each participant’s individual physical condition. For instance, Schiller *et al.* [19] and Endres *et al.* [22] categorized TTH based on TCM meridian theory, including Yangming, Taiyang, Shaoyang, and Jueyin meridians. Additionally, three studies used TCM diagnostic methods to identify specific syndromes and then selected acupoints based on these diagnoses [22, 24, 26]. In contrast, two studies provided only general descriptions of the acupoints used [23, 27].

Acupuncture was employed in eight studies [18, 19, 22, 23, 24, 27, 28, 29], electroacupuncture (EA) in three studies [20, 21, 26], and laser acupuncture (LA) in one study [25]. With the exception of White *et al.* [28], which did not specify the needle retention time, each acupuncture session generally lasted between 20 and 30 minutes and was administered over a period of 5 to 12 weeks. In most studies, the sessions were repeated for 6 to 8 weeks, with treatments taking place once or twice per week.

The results from 11 studies, excluding White *et al.* [28], demonstrate that acupuncture was effective for treating TTH without causing serious adverse effects. In addition, acupuncture was shown to improve the response rate and quality of life, reduce pain intensity as measured by visual analog scale (VAS) scores, and decrease pain duration. Follow-up assessments in most studies also indicated a sustained beneficial effect of acupuncture on TTH. Additionally, the study by Chassot *et al.* [20] reported that electroacupuncture not only reduced VAS scores and the need for analgesics in TTH participants but also increased serum levels of brain-derived neurotrophic factor (BDNF).

## 4. Discussion

According to a Cochrane systematic review, available evidence suggests that acupuncture is effective for treating frequent episodic or chronic TTH [30]. The European Federation of Neurological Societies guidelines also recognize acupuncture as a potentially important option for patients with frequent TTH [31]. However, previous meta-analyses have primarily demonstrated the efficacy of acupuncture for TTH without providing detailed explanations of its mechanisms.

Acupuncture, derived from TCM theory, is a complex, non-pharmacological intervention involving multiple interacting components [32]. Table 2 presents the results of current acupoint selection for TTH in modern clinical trials, reflecting the insights of ancient TCM practitioners. In TTH treatment, acupuncture prescriptions may vary due to individual differences among patients. Practitioners must possess a solid foundation in TCM and tailor treatment based on the patient’s specific symptoms, which accounts for the variability in mandatory and optional acupoints observed across the 12 studies listed in Table 2.

Chinese experts have noted that the efficacy of acupuncture for TTH is influenced by factors such as the selection of acupoints, frequency of sessions, duration of needle retention, and type of acupuncture used [33]. Furthermore, the methods for selecting acupoints are complex and require further exploration. These factors and their potential mechanisms warrant additional discussion to enhance our understanding of acupuncture’s role in treating TTH.

**Table 2.** Summary of the included studies.

References	No. of cases	Type	Mandatory Acupoints	Optional Acupoints	Intervention	Outcomes
Zheng *et al*. [18] 2022	110 (28 men; 82 women)	Chronic	Fengchi (GB 20), Baihui (GV 20), Taiyang, Hegu (LI 4), Taichong (LR 3)	Not reported	AC, 30 min, 20 sessions for 8 weeks, 3 sessions per week for the first 4 weeks, 2 sessions per week for the last 4 weeks	The responder rate (defined as a participant with a >50% reduction in monthly headache days after treatment) was 68.2% at both week 16 and week 32. The reduction in the monthly number of headache days was 13.1 ± 9.8 days at week 16 and 14 ± 10.5 days at week 32.
Schiller *et al*. [19] 2021	24 (6 men; 18 women)	Mixed	Baihui (GV 20), Taiyang, Fengchi (GB 20), Hegu (LI 4)	-Yangming meridian: Neiting (ST 44), Yintang -Shaoyang meridian: Zulinqi (GB 41), Waiguan (SJ 5) -Taiyang meridian: Kunlun (BL 60), Houxi (SI 3) -Jueyin meridian: Taichong (LR 3), Neiguan (PC 6), Sishencong	AC, 30 min, 12 sessions; 2 sessions per week for 6 weeks	Large effects were observed for acupuncture across all pain intensity parameters between baseline and 3 months.
Chassot *et al*. [20] 2015	17	Chronic	Superior and central area of the tip of the triangular fossa in the ear; Helix root (ear); Medial aspect of the splenius capitis and semispinalis capitis muscles (C1–C2 level); Lateral aspect of the trapezius; Semispinalis capitis muscle (C6–T1 level); Levator scapulae muscles (C6–T1 level); Abductor pollicis brevis and dorsal interossei muscles (hands)	Not reported	EA, 30 min, 10 sessions; 2 sessions per week for 5 weeks	EA was statistically significant in reducing VAS scores and the use of analgesics compared with baseline. Additionally, EA increased serum BDNF levels.
Wang *et al*. [21] 2007	18 (10 men; 8 women)	Chronic	Taiyang, Fengchi (GB 20), Hegu (LI 4)	Not reported	EA, 3 min for each acupoint, twice a day for 4 weeks	The pain duration was shortened at week 2, and pain intensity decreased at both week 2 and week 4 compared with baseline.
Endres *et al*. [22] 2007	209 (46 men; 163 women)	Mixed	Baihui (DU 20), Hegu (LI 4), Taichong (LR 3) Xingjian (LR 2), Fengchi (GB 20) Tianzhu (BL 10)	Acupoints were selected based on different criteria: For painful areas, Ahshi points were used to address tender spots directly associated with pain. When focusing on regions of the head according to channels, the Shaoyang channel included Yangbai (GB 14), Shuaigu (GB 8), Fengchi (GB 20), Taiyang, Waiguan (SJ 5), and Zulinqi (GB 41). The Yangming channel encompassed Touwei (ST 8), Yintang, Shangxing (DU 23), Hegu (LI 4), and Neiting (ST 44). For the Taiyang channel, acupoints such as Cuanzhu (BL 2), Tianzhu (BL 10), Fengfu (DU 16), Dazhui (DU 14), Houxi (SI 3), Kunlun (BL 60), and Lieque (LU 7) were considered. The Jueyin channel points included Baihui (DU 20), Sishencong, Taichong (LR 3), and Neiguan (PC 6). In terms of internal Chinese syndromes, Liver Qi Stagnation was addressed with Taichong (LR 3), Ganshu (BL 18), Yanglingquan (GB 34), and Danzhong (RN 17). Liver Yang Rising was treated using Taichong (LR 3), Zulinqi (GB 41), and Yanglingquan (GB 34). Liver Fire Rising was managed with Xingjian (LR 2), Yangfu (GB 38), Neiting (ST 44), and Zulinqi (GB 41). Phlegm Retention was addressed through Fenglong (ST 40), Zhongwan (RN 12), Pishu (BL 20), Taibai (SP 3), and Yinlingquan (SP 9). For Spleen Qi Deficiency, the acupoints were Zusanli (ST 36), Neiguan (SP 6), and Qihai (RN 6). Liver Blood Deficiency was treated with Xuehai (SP 10), Ququan (LR 8), and Juque (RN 14). Kidney Yin Deficiency was managed with Guanyuan (RN 4), Fuliu (KI 7), and Sanyinjiao (SP 6). Kidney Yang Deficiency was addressed using Taixi (KI 3) and Shenshu (BL 23). For symptomatic conditions, Jianjing (GB 21) was used for neck and cervical spine issues, Neiguan (PC 6) for nausea, and Yanglingquan (GB 34) for muscular tension.	AC, 30 min, 10 sessions; preferably 2 sessions per week in 6 weeks	At 6 months, 33% of patients were classified as responders (response defined as a reduction of more than 50% in headache days per month and no use of excluded concomitant medication or other therapies). Headache scores decreased rapidly, reaching approximately half of baseline levels within 6 weeks of the start of treatment, and remained low at the 6-month follow-up. Significant differences were observed between the acupuncture (AC) group and the sham acupuncture group in terms of headache days (*p* = 0.002), global assessment (*p* = 0.009), and treatment success (*p* = 0.002).
Söderberg *et al*. [23] 2006	30 (7 men; 23 women)	Chronic	Fengchi (GB 20), Yangbai (GB 14), Hegu (LI 4), Neiting (ST 44)	Optional acupoints included Neiguan (PC 6), Daling (PC 7), Sanyinjiao (SP 6), Yanglingquan (GB 34), Touwei (ST 8), *etc*.	AC, 30 min, 10–12 sessions in 10–12 weeks	Headache intensity decreased significantly at both 3 and 6 months after the last treatment compared with baseline.
Melchart *et al*. [24] 2005	132 (37 men; 95 women)	Mixed	Fengchi (GB 20), Jianjing (GB 21), Taichong (LR 3)	For headache management based on specific characteristics: -For mainly frontal headaches, the acupoints Hegu (LI 4), Shangxing (DU 23), Yintang, Taiyang, Neiting (ST 44), and Tinghui (GB 2) were used. -For headaches primarily located in the vertex, Baihui (DU 20) or Shangxing (DU 23) and Sishencong were selected. -For mainly neck pain, acupoints such as Tianzhu (BL 10), Kunlun (BL 60), Shenmai (BL 62), Dazhui (DU 14), Houding (DU 19), Houxi (SI 3), or Yanglao (SI 6) were considered appropriate. -For holocephalic pain with fatigue, extra point Taiyang, Sanyinjiao (SP 6), Yinlingquan (SP 9), Zusanli (ST 36), Fenglong (ST 40), and Zhongwan (RN 12) were recommended. -For headaches that worsen with wet or cold weather, Hegu (LI 4), Dazhui (DU 14), Shangguan (GB 3), Zhigou (SJ 6), and Xuanzhong (GB 39) were indicated. -For modalities of wind, dampness, or cold, use Hegu (LI 4), Dazhui (DU 14), Zhigou (SJ 6), and Yanglingquan (GB 34). -For modalities of cold or wind, the acupoints Hegu (LI 4), Lieque (LU 7), Waiguan (SJ 5), and Dazhui (DU 14) were recommended.	AC, 30 min, 12 sessions in 8 weeks (2 sessions per week for the first 4 weeks, followed by 1 session per week in the remaining 4 weeks)	The number of days with headache decreased by 7.2 days, and the proportion of responders, defined as those with at least a 50% reduction in headache days, was 46%.
Ebneshahidi *et al*. [25] 2005	25 (5 men; 20 women)	Chronic	Yangbai (GB 14), Fengchi (GB 20), Hegu (LI 4), Lieque (LU 7)	Not reported	LA, 43 seconds, 1.3 J (~13 J/cm^2^) for each acupoint, 10 sessions, 3 sessions per week	There were significant differences (*p* < 0.001) in changes from baseline in months one, two, and three for the median score of headache intensity, median duration of attacks, and median number of days with headache per month.
Xue *et al*. [26] 2004	20 (7 men; 13 women)	Mixed	Not reported	-External wind and phlegm: Hegu (LI 4), Waiguan (SJ 5), Fenglong (ST 40), and Kunlun (BL 60). -Blood stasis: Taichong (LR 3), Xingjian (LR 2), Hegu (LI 4), and Lieque (LU 7). -Deficiency of kidney Jing, Qi and blood: Sanyinjiao (SP 6), Zusanli (ST 36), Hegu (LI 4), and Waiguan (SJ 5). -Hyperactive yang of liver: Taichong (LR 3), Hegu (LI 4), Taixi (KI 3), and Waiguan (SJ 5).	EA, 30 min, 2 sessions per week for 4 weeks	Headache frequency, duration, and intensity; pain threshold; and quality of life improved after acupuncture on the distal acupoints. Although the effect diminished after a 3-month follow-up, there was still an improvement compared with the baseline assessment.
Karst *et al*. [27] 2001	34 (17 men; 17 women)	Mixed	Fengchi (GB 20), Hegu (LI 4), Taichong (LR 3)	Acupoints were selected depending on the symptoms: -Shuaigu (GB 8), Yangbai (GB 14), Jianjing (GB 21), Zulinqi (GB 41) for headache and related symptoms. -Cuanzhu (BL 2), Tianzhu (BL 10), Kunlun (BL 60) for neck pain and discomfort. -Lieque (LU 7), Waiguan (SJ 5) for upper respiratory and related symptoms. -Touwei (ST 8), Zusanli (ST 36), Neiting (ST 44) for gastrointestinal and systemic issues. -Baihui (DU 20) and Yintang for general well-being and central issues.	AC, 30 min, 2 sessions per week for 5 weeks	There was a significant but weak improvement in quality of life parameters, including clinical global impressions and the Nottingham Health Profile.
White *et al*. [28] 2000	25 (7 men; 18 women)	Chronic	Fengchi (GB 20), Hegu (LI 4)	Acupoints were chosen based on tenderness and the patient’s symptoms.	AC, no needle retention time for 6 weeks	No significant differences were found for days with headache per week, headache duration, or headache severity at any time point.
Tavola *et al*. [29] 1992	15 (2 men; 13 women)	Mixed	6–10 stainless steel needles placed in the head, hands and feet	Not reported	AC, 20 min, once a week for 8 weeks	At 1 month after the end of treatment and at the 12-month follow-up, the frequency of headache episodes, analgesic consumption, and the headache index significantly decreased over time compared to baseline. However, there were no significant changes in the duration or intensity of headache episodes.

*AC: acupuncture; EA: electroacupuncture; LA: laser acupuncture; BDNF: brain-derived neurotrophic factor; VAS: visual analog scale.*

### 4.1 Selection of acupoints

#### 4.1.1 Selection of acupoints based on TCM syndrome differentiation

Acupoints are selected according to TCM theory and their efficacy in addressing specific systemic symptoms or diseases [34]. In TTH, the headache may present in varying locations or have no fixed site, and it may be accompanied by symptoms such as lumbar pain, lower limb weakness, and memory loss. From a TCM perspective, these symptoms suggest abnormalities in the function of internal organs. Therefore, it is essential to use TCM prescriptions to categorize the condition into different TCM syndrome types. For instance, Xue *et al.* [26] classified TTH into several categories, including External Wind and Phlegm, Blood Stasis, Deficiency of Kidney Jing, Qi and Blood, and Hyperactive Yang of the Liver. Similarly, Endres *et al.* [22] categorized TTH into Liver Qi Stagnation, Liver Yang Rising, Liver Fire Rising, Phlegm Retention, Liver Blood Deficiency, and Kidney Yin Deficiency. Each category has distinct symptomatology, which is determined by analyzing the patient’s specific symptoms and manifestations.

By integrating the patient’s symptoms and overall condition, the clinician can accurately classify the patient into a TCM syndrome type and select the most appropriate acupoints for treatment [35]. This approach ensures that the treatment is tailored to the patient’s physical condition.

#### 4.1.2 Local acupoint selection

Local acupoint selection is a method where acupoints are chosen based on their proximity to the area affected by the disease or pain. This approach aligns with the principle of “pain as loss” described in the Yellow Emperor’s Classic of Internal Medicine. In our study, the local acupoints selected included Fengchi (GB 20), Baihui (GV 20), Taiyang, and Yangbai (GB 14), among others. These acupoints are primarily located on the head and are frequently used in this review.

Additionally, the Ahshi acupoint was utilized in the study by Endres *et al*. [22]. Ahshi points in TCM are identified as sensitive or reactive areas associated with the disease and are used as sites for acupuncture treatment [36]. According to TCM, the disease causes a blockage of qi and blood in specific body areas, leading to a localized accumulation of qi and blood, which results in Ahshi points. These points are significantly correlated with myofascial trigger points (TrPs). Both TrPs and Ahshi acupoints share pathological characteristics and are frequently employed in pain management. These points do not have fixed anatomical locations and typically elicit pain when pressure is applied [37].

### 4.2 Acupoint selection of corresponding meridian distal to the focus

Distal acupoint selection involves choosing acupoints located away from the primary lesion, reflecting the meridian theory of TCM [38]. As shown in Table 2, among the mandatory acupoints, Taichong (LR 3) and Xingjian (LR 2) are located on the foot and correspond to the foot-Shaoyin liver meridian, while Hegu (LI 4) is located on the hand and corresponds to the hand-Yangming large intestine meridian. According to TCM meridian theory, the pathways of both the foot-Jueyin liver meridian and the hand-Yangming large intestine meridian extend through the head. Both Schiller *et al*. [19] and Endres *et al*. [22] utilized TCM meridian theory in their selection of optional acupoints. They aligned the specific headache locations with the corresponding meridian pathways in TCM, categorizing them into Yangming meridian headache, Shaoyang meridian headache, Taiyang headache and Jueyin meridian headache. Acupoints corresponding to these meridians were selected for treatment. In practice, distal acupoints are often used in conjunction with local acupoints, guided by meridian theory (Fig. 2).

**Fig. 2.** Local Acupoint Selection and Distal Acupoint Selection Based on Meridian Theory. Note: The meridian theory of TCM categorizes headaches into Taiyang meridian, Shaoyang meridian, Yangming meridian, and Jueyin meridian headaches. The four red areas represent the regions covered by the corresponding meridians. (Created with Biorender.com ).

### 4.3 Types of acupuncture

Electroacupuncture is a treatment method that combines traditional acupuncture with electric stimulation. This technique involves applying trace pulsed currents to acupuncture needles, which are similar to the body’s bioelectricity. The stimulation aims to trigger a systemic reflex mechanism through gentle nervous system activation [39]. Current research indicates that electroacupuncture can modulate various cell signal transduction pathways, thereby reducing neuroinflammation in animal models [40]. Xue *et al*. [26] demonstrated that electroacupuncture effectively decreases the frequency, duration and intensity of TTH and enhances quality of life. However, some clinical studies involving electroacupuncture lack detailed descriptions of the stimulation frequency or the rationale for its selection [20, 21]. Previous studies [41, 42] have suggested that a frequency of 2 Hz stimulates the release of enkephalins and endomorphins, while 100 Hz triggers the release of dynorphins. For optimal effects, a sparse-dense wave combination of 2/100 Hz is recommended. However, the comparative effects of endorphins and dynorphins in TTH patients have not been validated.

Laser acupuncture, as discussed by Ebneshahidi *et al*. [25], utilizes a low-intensity laser beam to directly irradiate acupoints. This method is painless, non-invasive, and does not require the use of complex instruments, unlike traditional acupuncture. Despite its advantages, the mechanism of laser acupuncture remains unclear, and many researchers emphasize that the efficacy of laser acupuncture is significantly influenced by the correct selection of acupoints [43].

### 4.4 Treatment duration

The duration of acupuncture treatment is influenced by various factors, including the type of disease, the season of onset, the disease’s duration and the patient’s overall physical condition. Clinicians often base the treatment duration on their experience and the patient’s availability. For instance, patients with a long-standing history of TTH typically require a more extended treatment period. As shown in Table 2, most studies employed treatment courses lasting more than five weeks, which may reflect the cumulative effects of acupuncture. This cumulative effect is thought to arise from the brain’s ability to “remember” the acupuncture signals, with repeated stimulation enhancing the consolidation and encoding of these signals. Additionally, acupuncture can provide immediate relief in pain management; for example, a single session of acupuncture can alleviate pain in patients experiencing an acute headache attack [44, 45].

### 4.5 Duration of needle retention

In Table 2, most clinical trials for headache treatment set the needle retention time to 20–30 minutes per session. However, there are no established standards or guidelines for needle retention times specifically for TTH treatment. A qualitative and quantitative analysis has identified needle retention time as a crucial factor influencing the efficacy of acupuncture for TTH [46]. It has been suggested that an optimal needle retention time for TTH is 30 minutes [46]. White *et al.* [28] reported that acupuncture did not significantly improve the number of headache days per week, headache duration, or severity, and this study did not specify needle retention times. Conversely, Shi *et al.* [47] conducted a study on vascular TTH and found that longer needle retention times (30 and 40 minutes) resulted in lower scores on the 6-point behavioral rating scale compared to shorter retention times (10 and 20 minutes), with statistically significant differences (*p* < 0.05).

### 4.6 Related mechanisms

When specific areas of skeletal muscle are not adequately relaxed, they can become tense and hypersensitive to pressure, forming what are known as trigger points (TrPs). These TrPs are frequently associated with chronic TTH and are commonly located in the biting, temporal, sternocleidomastoid and trapezius muscles [48]. Persistent low-level muscle activity can lead to muscle fiber damage, which results in the accumulation of analgesic factors such as 5-hydroxytryptamine, calcitonin gene-related peptide, and substance P in the affected region. These factors may stimulate peripheral pain receptors, initiating a pain response that is transmitted to the central nervous system [49]. Over time, this persistent pain signal can impair nociceptive regulatory mechanisms, contributing to the chronic nature of TTH. In our study, many frequently used acupoints were located in areas corresponding to TrPs in the pericranial muscles. For example, Touwei (ST 8) and Taiyang are situated in the regions associated with the jaw and temporal muscles, while Fengchi (GB 20) is located between the sternocleidomastoid and trapezius muscles. Activation of these acupoints may help alleviate TrPs and reduce associated pain.

Headaches can lead to central sensitization, which ultimately results in nociceptive sensitization [50, 51]. Acupuncture may influence this central sensitization pathway, potentially mitigating its effects [52, 53]. Animal studies have demonstrated that electroacupuncture can enhance the release of endogenous opioid peptides [54]. Additionally, acupuncture at the local level can reduce the levels of inflammatory mediators and increase the presence of local endorphins and peripheral opioid receptors in response to inflammation [55, 56]. Elevated serum levels of BDNF in the cerebrospinal fluid have been associated with enhanced synaptic plasticity in rats [57, 58]. This increased synaptic plasticity and the accumulation of neurotransmitters, such as substance P and glutamate, contribute to prolonged sensitization of headache centers [59, 60]. A clinical trial has shown that electroacupuncture can inhibit this pathway and decrease neuroplasticity by reducing BDNF levels [18].

## 5. Limitation

Although this review aimed to explore the role of acupuncture in treating TTH from the perspective of TCM and related mechanisms, several limitations must be acknowledged. First, the study is constrained by a small sample size and overall low quality of evidence, which may affect the reliability of the conclusions. Second, there is significant variability in acupoint selection due to differing diagnostic approaches among studies. This lack of consensus on effective acupoints complicates efforts to determine the most beneficial acupoints for TTH treatment. Third, there was a lack of research examining the quantitative effects of acupuncture sessions, the duration of needle retention, and the comparative efficacy of different types of acupuncture. Fourth, there is also a lack of large-sample, multicenter, high-quality clinical studies, leading to generally low methodological and evidence quality. Fifth, the absence of standardized guidelines for acupuncture treatment of TTH further complicates clinical practice. Lastly, acupuncture for TTH remains relatively understudied in terms of its mechanisms, which necessitates further investigation in future research.

## 6. Conclusion

This review represents the first attempt to analyze the therapeutic role of acupuncture in treating TTH from both TCM perspectives and potential underlying mechanisms. It provides a foundation for future research and offers practical insights for clinicians utilizing acupuncture to manage TTH. Based on the current evidence, a preliminary therapeutic regimen is suggested: First, acupuncturists should select acupoints based on both local and distal sites, guided by TCM syndrome differentiation and the individual characteristics of each patient. Second, the acupuncture treatment course should be extended for no less than four weeks to ensure efficacy. Third, each acupuncture session should last at least 20 minutes, with a recommended duration of 30 minutes to optimize therapeutic outcomes.

## 7. Key findings

Based on current literature, patients with TTH may benefit from acupuncture therapy. Key factors contributing to effective treatment include the precise selection of acupoints, the duration of needle retention, the type of acupuncture used, and the overall length of the treatment course. These elements are essential for optimizing therapeutic outcomes in TTH management.
